# Partners, Pride, and Prevention: Scaling Mpox Vaccination Access Across Minnesota

**DOI:** 10.3390/ijerph23050593

**Published:** 2026-04-30

**Authors:** Ingrid M. E. Johansen, Darcey K. McCampbell, Luke M. Leners

**Affiliations:** Community Advancement—Community Clinical Care, Fairview Health Services, Minneapolis, MN 55454, USA; ingrid.johansen@fairview.org (I.M.E.J.); darcey.mccampbell@fairview.org (D.K.M.)

**Keywords:** mpox vaccination, JYNNEOS vaccine, mobile clinics, culturally responsive outreach, rural outreach, health equity

## Abstract

**Highlights:**

**Public health relevance—How does this work relate to a public health issue?**

- Mpox is a vaccine-preventable infectious disease that caused a significant outbreak in the United States in 2022 which disproportionately impacted gay, bisexual, and other men who have sex with men (MSMs), people living with HIV, racial and ethnic minority communities, and residents of rural areas.
- This work addresses persistent public health challenges surrounding equitable access to vaccines, stigma, geography, and cultural competency by examining a real-world, community-based vaccination intervention as a component of the response to an emerging infectious disease outbreak.

**Public health significance—How is this work of significance to public health?**

- The following brief report demonstrates that a mobile, partnership-driven vaccination model can effectively align preventive resources with the needs of disproportionately impacted and historically marginalized communities, as evidenced by program-level estimates of vaccination-to-case ratios among American Indian/Alaska Native, Black, and Hispanic male participants.
- The findings highlight the integral role of trusted community settings—particularly Pride events—in creating avenues for initial engagement and creating an opportunity to address key gaps in outbreak vaccination strategies.

**Public health implications—What are the key implications or messages for practitioners, policy makers and/or researchers in public health?**

- For practitioners: Mobile clinics hosted in trusted and affirming community environments can reduce stigma, logistical barriers, and missed opportunities for prevention, particularly for LGBTQ+ populations, people of color, uninsured individuals, and rural or underserved communities.
- For policy makers: Sustained investment in flexible, community-centered vaccination infrastructure and facilitation of cross-functional partnerships is essential to proactively ensure equitable access to preventive health resources, especially in the context of infectious disease outbreak management and localized provider shortages.

**Abstract:**

Mpox is a rare but potentially serious vaccine-preventable disease. The 2022 United States outbreak disproportionately impacted gay, bisexual, and other men who have sex with men, people living with HIV, and people of transgender experience. Early vaccination efforts revealed substantial racial and geographic inequities, with lower uptake among Black and Hispanic cisgender men, transgender women, and residents of rural areas. To address these challenges, Fairview’s Minnesota Immunization Networking Initiative (MINI), a 20-year-old mobile health collaborative, partnered with state and local public health agencies and community-based organizations to expand mpox vaccine access. With support from governmental outbreak response funding and stockpiled vaccine, mobile clinics were deployed in trusted community settings, including Pride events and recurring community sites. Targeted outreach, education, and coordination with local providers supported stigma reduction and second-dose series completion. Program data were collected from October 2022 through December 2024. MINI hosted 125 community-based mpox vaccination events, administered 2259 doses to individuals from 220 cities across the United States, including 195 cities in the Midwest. Pride events were key entry points for first-dose vaccination, particularly in rural areas; urban non-Pride clinics played a complementary role in facilitating second-dose completion. Program-level vaccination-to-case ratios were highest among populations experiencing disproportionate mpox burden, including Black, Hispanic, and American Indian/Alaska Native male participants, suggesting alignment of preventive resources with community need. MINI’s mobile, partnership-driven approach demonstrates the value of pairing large-scale community events with recurring clinics to address barriers to both vaccine access and series completion. These findings underscore the importance of flexible, community-centered infrastructure in advancing health equity and strengthening outbreak preparedness.

## 1. The Challenge

Mpox, also referred to as “monkeypox,” is a rare but potentially serious, vaccine-preventable disease. In 2022, the United States (U.S.) experienced a significant outbreak with 29,939 confirmed cases, including 234 cases in Minnesota [1,2,3]. Although anyone can be exposed through close physical contact, the national outbreak disproportionately affected gay, bisexual, and other men who have sex with men (MSMs), as well as racial and ethnic minority groups [4,5].

These disparities mirror broader, longstanding health inequities experienced by lesbian, gay, bisexual, transgender, queer (LGBTQ+) communities. Discrimination, stigma, limited availability of culturally competent care, and structural inequities in employment, insurance coverage, and social support contribute to poorer health outcomes. These challenges are further shaped by the intersecting impacts of race, ethnicity, socioeconomic status, and geography, compounding barriers to care [6,7].

Data from the outbreak elucidated substantial inequities. Nationally, Black men had the lowest vaccination-to-case ratio (8.8), followed by Hispanic men (16.2), while white men had the highest ratio (42.5), indicating far greater barriers to vaccination for communities of color [1]. Similar disparities were communicated by local public health and community-based organizations in Minnesota, indicating that white cisgender men exhibited the highest vaccination rates, while uptake among Black and Latino cisgender men and transgender women was markedly lower.

The 2022 mpox outbreak demonstrated a critical need for innovative and culturally responsive public health strategies. Addressing the mismatch between populations most affected by mpox and those with the greatest access to vaccination requires tailored outreach, trusted community partnerships, and approaches that directly confront underlying inequities.

## 2. The Goal

Fairview, one of Minnesota’s largest nonprofit health systems, has served communities across the state for more than 100 years. With care sites spanning the twelve-county greater Minneapolis–St. Paul metropolitan region and extending statewide, Fairview occupies a central role in Minnesota’s healthcare landscape. In 2006, Fairview and several community partner organizations launched the Minnesota Immunization Networking Initiative (MINI)—a mobile collaborative dedicated to reducing vaccination barriers among populations experiencing health inequities [8]. MINI addresses access challenges by partnering closely with grassroots community organizations and local public health agencies to deliver free, mobile vaccination clinics in trusted community settings.

During the 2022 mpox outbreak, MINI’s longstanding community relationships and adaptable infrastructure enabled a rapid response, including targeted outreach and expanded vaccine accessibility. However, initial response planning exposed a notable absence of sufficiently developed partnerships centering care in the LGBTQ+ community. Addressing this gap was essential to implementing an effective, timely response and required that MINI quickly develop new partnerships and trust-building efforts within these communities. Guided by locally informed priorities and a commitment to culturally responsive care, MINI aimed to implement an outbreak response that expanded its network of partners serving the LGBTQ+ population, strengthened collaboration with public health agencies, deployed mobile vaccination clinics at Pride events and recurring community-based sites, conducted targeted outreach to populations at elevated risk, and facilitated connections to ongoing care for sustained access to vaccination.

## 3. The Execution

### 3.1. Growing the Network of Partners Serving LGBTQ+ Communities in Minnesota

At the onset of the outbreak, MINI conducted an inventory of existing partners supporting LBGTQ+ communities and recognized a need to grow this network. Essential to achieving this goal was collaboration with the MPLS Mpox Taskforce, a grassroots initiative which provided “a community response to a public health crisis” [9]. The collaboration began following a public forum moderated by the MPLS Mpox Taskforce and attended by MINI program leadership, eventually resulting in a new partnership with 35 organizations supporting LGBTQ+ communities.

### 3.2. Collaboration with State and Local Public Health

Since its inception, MINI has maintained close collaboration with the public health sector and has earned a reputation as a trusted agent among underserved communities [10]. During the mpox outbreak response, these relationships enabled early discussions about MINI’s capacity to reach populations beyond those served by brick-and-mortar healthcare alone. MINI subsequently applied for outbreak response grant funding through the Minnesota Department of Health (MDH) and became a listed provider of government-stockpiled mpox vaccines. MDH also supported outreach efforts (e.g., incentives for vaccination, community giveaways, provision of printed health resources) and connected MINI with local public health agencies and nonprofit organizations to troubleshoot vaccine access issues and disseminate requests for community-based clinic events.

### 3.3. Mobile Vaccination Clinics at Pride Events

In partnership with the MPLS Mpox Taskforce and other trusted organizations, MINI initiated free vaccination services at Pride events across Minnesota. Rooted in a history of protest and mutual care following the Stonewall uprising, Pride events, held throughout the Twin Cities metro and greater Minnesota, provided unparalleled opportunities to reach diverse populations in affirming, stigma-reducing settings.

The largest single mobile vaccination event provided by MINI was co-located with the Twin Cities Pride Festival, which has an annual attendance of over 500,000 individuals [11]. At this event, mpox vaccines were offered alongside other preventive health services (e.g., seasonal influenza vaccines, COVID-19 vaccines, blood pressure checks, oral health services, and hand massages) to enhance access to care and mitigate potential stigma associated with a single-service clinical event. In 2023, MINI vaccinated 311 individuals at the Twin Cities Pride Festival. In 2024, MINI independently vaccinated 286 individuals, while an additional 150 individuals received mpox vaccines from a partnering agency as part of a coordinated effort to increase on-site vaccine availability.

MINI leveraged rural Pride events to seed collaboration in areas where mpox vaccines were otherwise extremely limited and to coordinate second-dose referrals with local providers or, when no provider existed within 50 miles, to explore opportunities for return clinics. Mobile vaccination clinics were provided at newly founded and long-standing rural Pride events across Minnesota. This included the annual East Central Pride in Pine City, a town of approximately 3500 residents located an hour north of the Twin Cities metro, which is thought to be the oldest rural Pride event in the U.S. [12,13,14]. Other rural mobile vaccination clinics included Range Iron Pride in Virginia, Minnesota near the Canadian border and Alexandria Pride in the state’s west central region, situated approximately an hour from the South Dakota border. Within each town, the intersectionality of being part of LGBTQ+ and rural communities created unique barriers in access to care. Not all individuals felt able to receive mpox vaccination even when it was offered in their community. For example, during the Twin Cities Pride Festival, one attendee described traveling a considerable distance from their rural community and noted that concerns about being publicly identified as LGBTQ+ near their home would have precluded them from seeking vaccination, even if the vaccine had been locally available. Mobile vaccination clinics held in urban areas across the state, such as Duluth and Saint Paul, created opportunities to address these concerns and mitigate potential stigma.

### 3.4. Creation of Recurring Community-Based Vaccination Sites

To further increase access, mobile vaccination clinics were initiated at recurring sites within the Twin Cities, which prioritized communities experiencing homelessness and sexual exploitation. One such site in South Minneapolis, The Family Partnership, featured a collaboration between MINI, the MPLS Mpox Taskforce, the City of Minneapolis Health Department, MDH, and other social services agencies in the form of a monthly health fair.

### 3.5. Outreach to Communities at Risk

MINI’s key partner, the MPLS Mpox Taskforce, led a social media campaign to raise disease awareness and education about preventive measures, with a focus on Black, Indigenous, and People of Color (BIPOC), and LGBTQ+ Minnesotans. The MPLS Mpox Taskforce also maintained a dedicated website with information on testing, vaccination opportunities, and other health services [9]. The collaboration bridged partnerships with culturally specific community-based organizations, such as the African American AIDS Task Force, a Twin Cities nonprofit providing culturally specific prevention, education, and services to people of African descent living with or at risk for HIV [15]. The MPLS Mpox Taskforce also accompanied MINI vaccination teams to rural Pride events to support outreach efforts, increase disease awareness, and offer preventive education in rural communities.

### 3.6. Bridge to Care Through Second-Dose Vaccination Coordination

Optimal protection against mpox requires completion of a two-dose mpox (JYNNEOS) vaccine series [16]. Vaccine effectiveness is nearly twice as great for individuals who receive both doses (66%) compared to those who receive only a single dose (35.8%) [17]. To emphasize the importance of series completion and support continuity of care for the second dose, MINI and its partners delivered targeted health education at each mobile vaccination clinic and through the MPLS Mpox Taskforce website [9]. In addition, partnerships with local public health agencies helped establish referral sites for second-dose vaccination following Pride events, particularly in rural areas where access was limited. When no provider was available within 50 miles of a Pride event, MINI offered return mobile vaccination clinics to facilitate completion of the vaccine series.

Partners from external health systems also supported access to second-dose mpox vaccination. For example, at the 2024 St. Cloud Pride event, a large external health system serving central and southwestern Minnesota was situated adjacent to the MINI mpox vaccination clinic and helped to schedule second-dose vaccination appointments in primary care for community members who were provided an initial dose. The collaborative uniquely supported this opportunity via on-site referrals and reallocation of MINI’s mpox vaccines, sourced from the Strategic National Stockpile, as needed.

## 4. Hurdles

Efforts to increase mpox vaccination uptake were hindered by a range of structural, social, and operational challenges that collectively limited timely and equitable access to preventive services. These barriers were experienced most acutely by LGBTQ+ communities, Black and Latino individuals, and residents of rural areas—groups already disproportionately affected during the 2022 outbreak [1,5,18].

A significant challenge stemmed from low community awareness of mpox as a health threat. Although the virus has been documented in humans since 1970, the 2022 outbreak marked the first time many communities in the U.S. had encountered mpox as a local public health concern [19]. Limited familiarity with its symptoms, transmission dynamics, and vaccine recommendations contributed to confusion and reduced early vaccine uptake [20].

Stigma also played a substantial role [18]. Public discourse surrounding the virus, particularly early references to “monkeypox,” contributed to racialized and sexualized stigma that deterred many from seeking care [21]. While the World Health Organization later adopted the name mpox to mitigate these harms, fear of judgment, discrimination, and unintended disclosure of sexual orientation or behavior continued to discourage individuals from accessing both vaccination and treatment services [22].

Operational challenges further impeded access, particularly the scarcity of providers offering mpox vaccination. MINI frequently encountered discrepancies between publicly available information and actual provider availability, particularly within the CDC Vaccine Finder tool. Clinics listed as vaccination sites often no longer offered mpox vaccine or had never provided it, complicating the process of arranging second-dose referrals. In rural areas, some communities lacked any provider within a reasonable distance. Although MINI collaborated with local health departments and community partners to coordinate episodic vaccination opportunities at events such as Pride celebrations, these one-time clinics could not guarantee the consistent access required for two-dose series completion [17].

Geographic barriers were compounded by significant transportation challenges, particularly in rural communities. Studies indicate that rural residents traveled substantially farther and spent much more time accessing mpox vaccination compared to their urban counterparts, reflecting longstanding disparities in healthcare provider distribution [18]. These distances increased barriers—not only to initial vaccination but also to follow-up appointments.

Financial and policy shifts also shaped vaccine access. The commercialization of the mpox vaccine in April 2024 rendered it prohibitively expensive for many local public health agencies and limited its inclusion in provider formularies [23]. Large health systems frequently restricted vaccine access to infectious disease clinics concentrated in metropolitan areas, further diminishing availability, especially for communities outside the urban core [24]. This policy shift directly constrained MINI’s mobile vaccination capacity and reduced pathways for second-dose completion. During this period, MINI lacked the ability to bill private insurance, and state outbreak response funding was simultaneously winding down, resulting in the cessation of the mobile clinics. While these constraints limited vaccine delivery at the time, MINI has since invested in the operational infrastructure required to bill insurance, marking a critical progression toward financial sustainability and greater continuity of mobile vaccination services.

Finally, added operational and clinical barriers emerged following the Emergency Use Authorization (EUA) calling for intradermal vaccine administration [25]. While intradermal dosing extended limited vaccine supply by allowing more individuals to be vaccinated, it required clinical staff to learn a less commonly used administration technique [26]. Securing appropriate needles for intradermal administration posed additional logistical hurdles. Concerns also arose regarding the potential for keloid formation—particularly among individuals with darker skin tones—an issue not adequately examined in clinical trials due to the underrepresentation of Black and Hispanic participants [27,28]. This gap emphasized the need for more inclusive research and culturally responsive patient counseling to support informed, shared decision-making.

## 5. The Team

Volunteer-driven at its founding, MINI staffing has evolved, increasing proportionately along with the demand for community-based mobile vaccination clinics. Since its inception, funding for this initiative has been realized through a diversified approach that includes Fairview Foundation support, grants and contracts paired with a sustained commitment from Fairview’s community benefit investments. In 2018, MINI expanded its staffing model to include an interdisciplinary team of full-time and per diem professionals—consisting of registered nurses (RNs), an emergency medical technician (EMT), a paramedic, program registrars, and a program coordinator. Creation of the “community health nurse” role increased partner satisfaction through more consistent staffing and allowed for standardized clinical competencies, which optimized safe clinical practices. Additionally, the introduction of a program coordinator in 2019 improved operations management, fostering ongoing maintenance and expansion of community partnerships. This flexible staffing model, diversified funding, and institutional commitment have collectively allowed MINI to quickly respond to fluctuating and unpredictable immunization needs throughout the community, including the global COVID-19 pandemic, the 2019 Minnesota hepatitis A outbreak, and the 2022 U.S. mpox outbreak.

Across diverse community settings, baseline staffing for every vaccination clinic included two RNs and one program registrar; the number of RNs and registrars were scaled up according to anticipated attendance. Each clinic was coordinated in collaboration with a trusted community partner organization. Unique to the mpox outbreak response, MINI was supported at mobile vaccination clinics by auxiliary staff from the MPLS Mpox Taskforce, local public health services, or MDH. These colleagues brought expertise and navigation resources specific to the outbreak, often tailored to the particular community being served.

## 6. Metrics

From 8 October 2022 to 31 December 2024, the MINI team hosted 125 community-based mpox vaccination events and provided 2259 doses to recipients (see Table 1) from 220 different cities across the U.S., 195 of which were in the Midwest.

Recipient information was abstracted from vaccination records maintained on a Health Information Portability and Accountability Act (HIPAA)-compliant SharePoint site only accessible to MINI program staff. Records included all relevant demographic and vaccine information. All mpox vaccinations included in this analysis were administered at MINI partner sites located within the state of Minnesota; no vaccine doses provided outside Minnesota were included. Analysis of a limited dataset allowed for the determination of both recipients’ residential geography and the geography of clinical events. Residential geography was defined using the home address reported by recipients at the time of vaccination, which in some cases reflected addresses outside Minnesota. Factors considered in the geographic determination included city name, census tract, 2020 Rural–Urban Commuting Area (RUCA) codes, and population density metrics [29]. Addresses were ultimately grouped into three categories—urban, metro (i.e., suburban), and rural areas—and subsequently removed from the dataset.

After cessation of the mpox service offering, de-identified data were aggregated across all clinical events for the purpose of generating sample-level descriptive statistics, carrying out tabular analyses, and for comparison to statewide trends in mpox case incidence. The analytic dataset represents all vaccine doses administered during MIN’s mpox vaccination activities. Exclusion of recipients based solely on reported state of residence would have removed a non-random subset of participants and could have introduced bias into subgroup analyses, particularly by residential geography. Therefore, all recipients vaccinated at MINI events were retained in the primary analysis. Data were tabulated and simple descriptive statistical analyses (e.g., counts, percentages, and summary measures) were conducted using Microsoft^®^ Excel^®^ for Microsoft 365 (Version 2603). Permanent home address was self-reported and may not reflect temporary residence in Minnesota (e.g., students or seasonal workers). Temporary residential status was not systematically assessed and is noted as a limitation.

Of the 125 events, 89 (71.2%) occurred in urban settings, 31 (24.8%) occurred in metro settings, and 5 (4%) occurred in rural settings (see Table 2). By comparison, 1129 (50%) mpox doses were given to recipients who reported residing in an urban setting; 669 (29.6%) were given to recipients who reported residing in a metro setting; and 181 (8%) were given to recipients who reported residing in a rural setting (see Table 1).

An additional 280 (12.4%) mpox doses were given to recipients for which a determination of residential geography could not be made. Of all recipients with indeterminate residential geographies, 237 (84.6%) were vaccinated at urban events (see Table 1). This pattern may be indicative of privacy concerns, housing instability, or homelessness within this subgroup, especially considering multiple recurring urban events were held at locations providing targeted support to individuals experiencing homelessness and sexual exploitation. Missingness in classification of residential geography may have resulted in an incomplete or biased evaluation of participants’ residential proximity to vaccination sites. Regardless, across all three geographies, most recipients reported living in the same geographic classification as the clinical site where they were vaccinated: urban events tended to serve urban residents (*n*_Urban×Urban, Out of State Urban_ = 995, 57.1%); metro events tended to serve metro residents (*n*_Metro×Metro, Out of State Metro_ = 178, 46.6%); and rural events tended to serve rural residents (*n*_Rural×Rural, Out of State Rural_ = 79, 58.9%) (see Table 1). Although risk for mpox exposure and vaccine need existed throughout greater Minnesota, MINI’s observed operational footprint in and around urban centers reflected a paucity of partner organizations serving rural LGBTQ+ communities and the team’s necessary reliance on regional demand to drive event planning. Rural Pride events served as drivers of regional demand, disrupted sustained barriers to care in non-urban settings, and facilitated essential opportunities for initial engagement with community members seeking mpox education and vaccines.

22 (17.6%) mpox vaccination events coordinated by MINI took place at a Pride event; among Pride-affiliated events, 12 (54.5%) took place in urban geographies, 6 (27.3%) in metro geographies, and 4 (18.2%) in rural geographies (see Table 2). Just over half of all mpox vaccines were given at Pride events (*n*_Pride_ = 1231, 54.5%), highlighting their key function as high-volume and low-barrier settings for initial engagement (see Table 1). Despite the generally urban concentration of this work, nearly all vaccines administered at clinical sites located in rural geographies were provided at Pride-affiliated events (*n*_Rural×Pride_ *=* 131, 97.8%) (see Table 3) and to recipients who were also residents of rural areas (*n*_Rural×Rural, Out of State Rural_ = 79, 58.9%) (see Table 1). Most vaccines given at Pride events were patients’ first toward mpox series completion (*n*_Pride×First Dose Toward Series Completion_ = 1152, 93.6%) and this trend was conserved across all geographies, which suggests the important role played by non-Pride events in facilitating mpox series completion throughout the state (see Table 3). Urban non-Pride events served as the most representative locations for second-dose administration facilitated by MINI (*n*_Urban×Non-Pride × Second Dose (Series Completion)_ = 303, 17.4%) (see Table 3), demonstrating a complementary implementation strategy that paired Pride engagement efforts and recurring non-Pride events to best serve patients with complex barriers to access.

Across all events, MINI mpox vaccine clinic attendees tended to identify as males (*n*_Male_ = 1039, 46.0%), aged 19–34 (*n*_19–34_ *=* 1096, 48.5%), who were non-Hispanic, white (*n*_White_ = 1095, 48.5%), living in an urban area (*n*_Urban, Out of State Urban_ = 1129, 50%), and carrying active health insurance (*n*_Has Insurance_ = 1914, 84.7%) (see Table 1). Recipients were almost exclusively treated at clinical sites located in either urban or metro geographies (*n*_Urban, Metro_ = 2125, 94.1%) (see Table 1). Unsurprisingly, metro (suburban) residents exhibited the highest rates of insurance among all recipients (*n*_Has Insurance×All Metro Settings_ = 606, 90.6%), which remained consistent across nearly all reported racial and ethnic groups (see Table 4 and Table S1).

A more diverse representation of race and ethnicity was observed at non-Pride events, which included higher proportions of American Indian/Alaska Native (AI/AN), Black, and Hispanic recipients. Pride events attended by MINI, particularly those located in suburban and rural geographies, tended to draw predominantly non-Hispanic white attendees, shaping the observed demographic trends (see Table 3). A comparable number of transgender recipients were served at non-Pride (*n*_Non-Pride×Transgender—Male, Transgender—Female, Transgender—Unspecified_ = 56; 5.5%) and Pride (*n*_Pride×Transgender—Male, Transgender—Female, Transgender—Unspecified_ = 61; 5.0%) events across all geographies, indicative of success in MINI’s aim to create equitable intake or registration processes for community members regardless of gender identity or site of care (see Table 1).

Clinics hosted in urban geographies were more diverse, tended to include younger recipients ($\bar{x}$_Urban_ = 37 years, σ = 12.9; $\bar{x}$_Metro_ = 39.5, σ = 14.9; $\bar{x}$_Rural_ = 39.7, σ = 16.2) and had the highest rates of uninsurance among the three geographies (*n*_Urban×Uninsured_ = 235, 13.5%) (see Table 1). Importantly, AI/AN, Hispanic, and Black recipients who were also urban residents exhibited the highest rates of uninsurance among strata with appreciable sample sizes (*n*_All Urban Settings×AI/AN, non-Hispanic×Uninsured_ = 26, 19.2%; *n*_All Urban Settings×Hispanic×Uninsured_ = 16, 18.8%; *n*_All Urban Settings×Black, non-Hispanic×Uninsured_ = 43, 17.6%); each were more than double that observed among the population of urban, white recipients (*n*_All Urban Settings×White, non-Hispanic×*Uninsured*_ = 39, 8.1%) (see Table 4).

Disparities in insurance coverage among MINI mpox vaccine recipients mirrored those observed statewide, with Black, AI/AN, and Hispanic Minnesotans experiencing elevated uninsurance rates, suggestive of disrupted primary care access and delivery [30]. Heightened representation of these groups at urban non-Pride clinics and the key role served by non-Pride events in facilitating series completion are illustrative of MINI’s effective partnership with trusted community organizations to address gaps in care—especially those that may have been exacerbated by the mpox outbreak.

Incident mpox case data provided by MDH, combined with an adapted application of the methods described by Kota et al., were used to generate population-level estimates of mpox disease burden in Minnesota by race and ethnicity (see Table 5) [1].

These estimates indicate that, specifically among MSMs, mpox incidence rates were elevated in racial and ethnic minority groups—consistent with input from MINI’s community partners and trends reported elsewhere in the literature [1,4,5]. Over the duration of MINI’s mpox vaccine service offering, Black and Hispanic MSMs in Minnesota were estimated to have experienced the highest incidence rates relative to white MSMs (rate ratio (RR)_Black:White_ = 2.3; RR_Hispanic:White_ = 2.9) (see Table 5), reflecting disproportionate mpox disease burden in these communities. During the service timeframe, overall program utilization suggests that approximately 26 mpox vaccines were administered to male recipients by MINI for each incident case statewide (see Table 6).

Program-level vaccination-to-case ratios varied by race and ethnicity and were highest among AI/AN (97.6), Black (37.7), and Hispanic (27.0) male recipients (see Table 6). Although several limitations restrict the generalizability of these estimates—which should be interpreted as indicators of programmatic reach relative to disease burden rather than measures of population-level coverage—higher vaccination-to-case ratios among groups experiencing disproportionate mpox incidence suggest that MINI successfully aligned preventive resources with community need and advanced equitable access to mpox prevention through their mobile service offering.

## 7. Conclusions

The mpox outbreak highlighted deep structural and social inequities in access to prevention, care, and information, particularly for LGBTQ+ communities, people of color, individuals living with HIV, and rural residents. Barriers including limited awareness, stigma, provider shortages, transportation challenges, and vaccine commercialization underscored the difficulty of ensuring equitable vaccine access during an emerging infectious disease event. Against this backdrop, MINI implemented a powerful and adaptable community-based mobile care model. By partnering with local public health, community groups, and Pride organizers across the state, MINI directly delivered culturally responsive vaccination and health education in trusted settings. These efforts reached people where they already felt safe, reduced stigma-related barriers, and offered practical solutions in communities with few or no local providers.

The success of MINI’s approach—providing more than 2200 mpox vaccine doses to individuals from 195 cities across the Midwest, while delivering for those most at risk—demonstrates that mobile clinics are not only effective for rapid response, but essential to building equitable public health infrastructure. Meeting communities where they are, especially during times of heightened need, fosters trust, expands access, and helps ensure that prevention efforts reach those who are particularly vulnerable. As public health systems prepare for future outbreaks, lessons from MINI’s work accentuate the importance of flexibility, cultural responsiveness, and deep community partnership in the pursuit of equitable outbreak response.

## Figures and Tables

**Table 1:** Characteristics of MINI mpox vaccine recipients including distribution across clinical event site geography and Pride event affiliation.

Sample Characteristics	Total Recipients	Clinical Event/Site Geography			Pride Event Affiliation	
		Urban	Metro	Rural	Pride	Non-Pride
Demographics	(*n* = 2259)	(*n* = 1743)	(*n* = 382)	(*n* = 134)	(*n* = 1231)	(*n* = 1028)
Gender Identity						
Male	1039 (46.0%)	808 (46.3%)	190 (49.7%)	41 (30.6%)	537 (43.6%)	502 (48.8%)
Female	777 (34.4%)	574 (32.9%)	127 (33.2%)	76 (56.7%)	458 (37.2%)	319 (31.0%)
Transgender—Male	17 (0.8%)	15 (0.9%)	2 (0.5%)	0	7 (0.6%)	10 (1.0%)
Transgender—Female	11 (0.5%)	10 (0.6%)	1 (0.3%)	0	9 (0.7%)	2 (0.2%)
Transgender—Unspecified	89 (3.9%)	78 (4.5%)	11 (2.9%)	0	45 (3.7%)	44 (4.3%)
Non-Binary	71 (3.1%)	51 (2.9%)	11 (2.9%)	9 (6.7%)	51 (4.1%)	20 (1.9%)
Other/Not Listed	103 (4.5%)	82 (4.7%)	19 (5.0%)	2 (1.5%)	74 (6.0%)	28 (2.7%)
Unknown	152 (6.7%)	125 (7.2%)	21 (5.5%)	6 (4.5%)	50 (4.1%)	103 (10.0%)
Age, years (mean [SD])	37.6 [13.5]	37 [12.9]	39.5 [14.9]	39.7 [16.2]	35.5 [13.9]	40 [12.7]
Age group, years						
18	38 (1.7%)	22 (1.3%)	9 (2.4%)	7 (5.2%)	33 (2.7%)	5 (0.5%)
19–34	1096 (48.5%)	883 (50.6%)	159 (41.6%)	54 (40.3%)	673 (54.7%)	423 (41.1%)
35–44	528 (23.4%)	417 (23.9%)	88 (23.0%)	23 (17.2%)	252 (20.5%)	276 (26.8%)
45–54	305 (13.5%)	219 (12.6%)	60 (15.7%)	26 (19.4%)	137 (11.1%)	168 (16.3%)
55–64	187 (8.3%)	137 (7.9%)	37 (9.7%)	13 (9.7%)	76 (6.2%)	111 (10.8%)
65–74	90 (4.0%)	59 (3.4%)	26 (6.8%)	5 (3.7%)	47 (3.8%)	43 (4.2%)
75–84	15 (0.7%)	6 (0.3%)	3 (0.8%)	6 (4.5%)	13 (1.1%)	2 (0.2%)
Race/Ethnicity						
White, non-Hispanic	1095 (48.5%)	741 (42.5%)	262 (68.6%)	92 (68.7%)	798 (64.8%)	297 (28.9%)
Black, non-Hispanic	391 (17.3%)	348 (20.0%)	40 (10.5%)	3 (2.2%)	103 (8.4%)	288 (28.0%)
Hispanic	177 (7.8%)	139 (8.0%)	29 (7.6%)	9 (6.7%)	102 (8.3%)	75 (7.3%)
Asian, non-Hispanic	84 (3.7%)	70 (4.0%)	12 (3.1%)	2 (1.5%)	62 (5.0%)	22 (2.1%)
AI/AN, non-Hispanic	216 (9.6%)	198 (11.4%)	5 (1.3%)	13 (9.7%)	39 (3.2%)	177 (17.2%)
NH/PI, non-Hispanic	14 (0.6%)	11 (0.6%)	1 (0.3%)	2 (1.5%)	6 (0.5%)	8 (0.8%)
2+ Races, non-Hispanic	133 (5.9%)	107 (6.1%)	17 (4.4%)	9 (6.7%)	72 (5.8%)	61 (6.0%)
Other/Not Listed, Unknown	149 (6.6%)	129 (7.4%)	16 (4.2%)	4 (3.0%)	49 (4.0%)	100 (9.7%)
Residential Geography						
Urban	1105 (48.9%)	975 (55.9%)	123 (32.2%)	7 (5.2%)	438 (35.6%)	667 (64.9%)
Out of State Urban	24 (1.1%)	20 (1.2%)	3 (0.8%)	1 (0.7%)	17 (1.4%)	7 (0.7%)
Metro	622 (27.5%)	429 (24.6%)	158 (41.4%)	35 (26.1%)	479 (38.9%)	143 (13.9%)
Out of State Metro	47 (2.1%)	25 (1.4%)	20 (5.2%)	2 (1.5%)	42 (3.4%)	5 (0.5%)
Rural	146 (6.5%)	30 (1.7%)	38 (10.0%)	78 (58.2%)	138 (11.2%)	8 (0.8%)
Out of State Rural	35 (1.5%)	27 (1.6%)	7 (1.8%)	1 (0.7%)	31 (2.5%)	4 (0.4%)
Missing/Indeterminate	280 (12.4%)	237 (13.6%)	33 (8.6%)	10 (7.5%)	86 (7.0%)	194 (18.9%)
Health Insurance Status						
Has Insurance	1914 (84.7%)	1454 (83.4%)	343 (89.8%)	117 (87.3%)	1094 (88.9%)	820 (79.8%)
Uninsured	281 (12.5%)	235 (13.5%)	29 (7.6%)	17 (12.7%)	120 (9.7%)	161 (15.6%)
Unknown	64 (2.8%)	54 (3.1%)	10 (2.6%)	0	17 (1.4%)	47 (4.6%)

**Table 2:** MINI mpox vaccine clinical event by site geography and Pride affiliation.

Clinical Event/Site Geography	Total Events (*n* = 125)	Pride (*n* = 22)	Non-Pride (*n* = 103)
Urban	89 (71.2%)	12 (54.5%)	77 (74.7%)
Metro	31 (24.8%)	6 (27.3%)	25 (24.3%)
Rural	5 (4.0%)	4 (18.2%)	1 (1.0%)

**Table 3:** MINI Mpox vaccinations by series completion, clinical event site geography, and Pride affiliation.

Clinical Event/Site Geography	First Dose Toward Series Completion			Second Dose (Series Completion)		
	Total (*n* = 1893)	Pride (*n* = 1152)	Non-Pride (*n* = 741)	Total (*n* = 365)	Pride (*n* = 79)	Non-Pride (*n* = 286)
Urban Event × Race/Ethnicity	1439	763	676 *	303	57	246
White, non-Hispanic	602	443 (58.1%)	159 (23.5%)	139	37 (64.9%)	102 (41.5%)
Black, non-Hispanic	294	90 (11.8%)	204 (30.2%)	54	7 (12.3%)	47 (19.1%)
Hispanic	113	68 (8.9%)	45 (6.6%)	26	6 (10.5%)	20 (8.1%)
Asian, non-Hispanic	59	49 (6.4%)	10 (1.5%)	11	4 (7.0%)	7 (2.8%)
AI/AN, non-Hispanic	165	23 (3.0%)	142 (21.0%)	32	0	32 (13.0%)
NH/PI, non-Hispanic	8	4 (0.5%)	4 (0.6%)	3	0	3 (1.2%)
2+ Races, non-Hispanic	94	51 (6.7%)	43 (6.4%)	13	1 (1.8%)	12 (4.9%)
Other/Not Listed, Unknown	104	35 (4.6%)	69 (10.2%)	25	2 (3.5%)	23 (9.4%)
Metro Event × Race/Ethnicity	328	263	65	54	17	37
White, non-Hispanic	229	215 (81.8%)	14 (21.5%)	33	14 (82.3%)	19 (51.4%)
Black, non-Hispanic	29	3 (1.1%)	26 (40.0%)	11	0	11 (29.7%)
Hispanic	28	19 (7.2%)	9 (13.9%)	1	0	1 (2.7%)
Asian, non-Hispanic	8	5 (1.9%)	3 (4.6%)	4	2 (11.8%)	2 (5.4.%)
AI/AN, non-Hispanic	4	3 (1.1%)	1 (1.5%)	1	0	1 (2.7%)
NH/PI, non-Hispanic	0	0	0	1	0	1 (2.7%)
2+ Races, non-Hispanic	15	11 (4.2%)	4 (6.2%)	2	0	2 (5.4%)
Other/Not Listed, Unknown	15	7 (2.7%)	8 (12.3%)	1	1 (5.9%)	0
Rural Event × Race/Ethnicity	126	126	0	8	5	3
White, non-Hispanic	85	85 (67.5%)	0	7	4 (80.0%)	3 (100%)
Black, non-Hispanic	3	3 (2.4%)	0	0	0	0
Hispanic	9	9 (7.1%)	0	0	0	0
Asian, non-Hispanic	2	2 (1.6%)	0	0	0	0
AI/AN, non-Hispanic	13	13 (10.3%)	0	0	0	0
NH/PI, non-Hispanic	2	2 (1.6%)	0	0	0	0
2+ Races, non-Hispanic	8	8 (6.3%)	0	1	1 (20.0%)	0
Other/Not Listed, Unknown	4	4 (3.2%)	0	0	0	0

* *n* missing or unknown = 1 (was given at an urban, non-Pride-affiliated event).

**Table 4:** MINI Mpox vaccine recipients’ insurance status by residential geography and reported racial/ethnic background.

Residential Geography	Total Recipients (*n* = 1979)	Has Insurance (*n* = 1715)	Uninsured (*n* = 215)	Unknown (*n* = 49)
All Urban Settings × Race/Ethnicity	1129	950	144	35
White, non-Hispanic	483	440 (91.1%)	39 (8.1%)	4 (0.8%)
Black, non-Hispanic	244	188 (77.1%)	43 (17.6%)	13 (5.3%)
Hispanic	85	67 (78.8%)	16 (18.8%)	2 (2.4%)
Asian, non-Hispanic	26	22 (84.6%)	4 (15.4%)	0
AI/AN, non-Hispanic	135	105 (77.8%)	26 (19.2%)	4 (3.0%)
NH/PI, non-Hispanic	5	5 (100%)	0	0
2+ Races, non-Hispanic	67	63 (94.0%)	4 (6.0%)	0
Other/Not Listed, Unknown	84	60 (71.4%)	12 (14.3%)	12 (14.3%)
All Metro Settings × Race/Ethnicity	669	606	51	12
White, non-Hispanic	412	381 (92.5%)	29 (7.0%)	2 (0.5%)
Black, non-Hispanic	61	52 (85.2%)	5 (8.2%)	4 (6.6%)
Hispanic	59	51 (86.4%)	6 (10.2%)	2 (3.4%)
Asian, non-Hispanic	48	45 (93.7%)	2 (4.2%)	1 (2.1%)
AI/AN, non-Hispanic	19	18 (94.7%)	0	1 (5.3%)
NH/PI, non-Hispanic	2	1 (50.0%)	1 (50.0%)	0
2+ Races, non-Hispanic	37	33 (89.2%)	4 (10.8%)	0
Other/Not Listed, Unknown	31	25 (80.6%)	4 (12.9%)	2 (6.5%)
All Rural Settings × Race/Ethnicity	181	159	20	2
White, non-Hispanic	131	118 (90.1%)	12 (9.2%)	1 (0.7%)
Black, non-Hispanic	5	4 (80.0%)	1 (20.0%)	0
Hispanic	11	6 (54.5%)	5 (45.5%)	0
Asian, non-Hispanic	3	2 (66.7%)	1 (33.3%)	0
AI/AN, non-Hispanic	14	12 (85.7%)	1 (7.1%)	1 (7.1%)
NH/PI, non-Hispanic	1	1 (100%)	0	0
2+ Races, non-Hispanic	11	11 (100%)	0	0
Other/Not Listed, Unknown	5	5 (100%)	0	0

Data for 280 immunizations are not included here due to indeterminate residential areas.

**Table 5:** Estimated statewide mpox disease burden and disparate trends by race/ethnicity in Minnesota from 8 October 2022 to 31 December 2024.

Race/Ethnicity	Estimated Population ^a^	Estimated MSMs ^b^	Incident Cases	Incident Cases per 100,000 MSMs ^d^	Rate Ratio (Ref. White)
White	4,402,795	171,709	31	18.5	-
Black	475,038	18,526	8	43.2	2.3
Hispanic **^c^**	388,141	15,137	8	52.9	2.9
Asian	330,210	12,878	3	23.3	1.3
AI/AN	86,897	3389	1	29.5	1.6

^a^ United States Census Bureau data were referenced to produce population estimates by race and ethnicity based on the proportionate representation of each group statewide. Estimated counts were rounded to the nearest whole number. ^b^ The estimated number of gay, bisexual, and other men who have sex with men (MSMs) for each subgroup was taken to be 3.9% of the total statewide population, consistent with the method employed by Kota et al. in generating national estimates [1]. **^c^** There were 50 total incident cases reported during the service timeframe according to MDH. Race and ethnicity were evaluated separately by MDH, such that individuals within each racial subgroup may have also identified as Hispanic. All reported incident cases by race are presented above and treated as if they were non-Hispanic; arbitrary removal of cases from racial subgroups was avoided in the interest of preserving trends in the data. ^d^ Case data by MSM status were unavailable and not monitored by MINI. As such, the incidence rates among MSMs and resultant rate ratios reflect an assumption that all observed cases in men during the service timeframe impacted MSMs.

**Table 6:** Mpox incidence in Minnesota and estimated MINI program-level vaccination-to-case ratios among adult male recipients by race/ethnicity from 8 October 2022 to 31 December 2024.

Characteristics	Incident Cases	Estimated Male Cases ^b^	MINI Mpox Vaccines	Male Recipients of MINI Mpox Vaccine ^c^	Vaccination-to-Case Ratio Among Male Recipients ^d^
Race					
White	31 (62.0%)	25.42 [21.7–27.9]	1095 (48.5%)	632 (28.0%)	24.9 [22.7–29.1]
Black	8 (16.0%)	6.56 [5.6–7.2]	391 (17.3%)	247 (10.9%)	37.7 [34.3–44.1]
Asian	3 (6.0%)	2.46 [2.1–2.7]	84 (3.7%)	42 (1.9%)	17.1 [15.6–20.0]
AI/AN	1 (2.0%)	0.82 [0.7–0.9]	216 (9.6%)	80 (3.5%)	97.6 [88.9–114.3]
Unknown	7 (14.0%)	5.74 [4.9–6.3]	149 (6.6%)	44 (1.9%)	7.7 [7.0–9.0]
Hispanic Ethnicity					
Hispanic **^a^**	8 (16.0%)	6.56 [5.6–7.2]	177 (7.8%)	76 (3.4%)	27.0 [24.6–31.6]
Non-Hispanic	35 (70.0%)	28.70 [24.5–31.5]	1941 (86.0%)	942 (41.7%)	26.9 [29.9–38.4]
Unknown	7 (14.0%)	5.74 [4.9–6.3]	141 (6.2%)	38 (1.7%)	5.4 [6.0–7.5]
*Overall Trend*	50	41	2259	1056	25.8

^a^ all participants who indicated their ethnicity to be “Hispanic/Latinx” on program consent forms were counted as Hispanic. Participants that indicated they “Chose not to respond,” or left the field blank were counted as Unknown. ^b^ Again, aggregated MDH case data made assessment of the true number of male cases by race ethnicity impossible. Sensitivity analyses were performed, with point estimates assuming that 82 percent of cases occurred in males (chosen because this was the cumulative proportion of cases among males and transgender males across race and ethnicity reported to MDH), the lower bound assuming 70 percent and the upper bound assuming 90 percent. Small case numbers among some subgroups and the generation of expected counts in the sensitivity analyses indicated the use of fractional case estimates to avoid distortion from rounding. ^c^ In keeping with the process for generating expected case estimates, MINI vaccine recipients who were male or transgender male were regarded as male recipients of MINI Mpox vaccines. ^d^ Program vaccination-to-case ratios are not reflective of vaccine effectiveness in relevant subgroups. They contextualize programmatic vaccination activity related to estimated mpox burden within the population served.

## Data Availability

The datasets presented in this article are not readily available because of protected health information and the sensitive nature of the topic. Requests to access the datasets should be directed to the corresponding author on reasonable request.

## Supplementary Materials

The following supporting information can be downloaded at: https://www.mdpi.com/article/10.3390/ijerph23050593/s1, Table S1: MINI Mpox vaccine recipients’ insurance status by residential geography and clinical event Pride affiliation.
