# Supplementary material for: Partners, Pride, and Prevention: Scaling Mpox Vaccination Access Across Minnesota
Source: Int J Environ Res Public Health. 2026 Apr 30;23(5):593. doi: 10.3390/ijerph23050593 (PMC13206966; doi:10.3390/ijerph23050593)
Supplement: Supplementary file 1 [file ijerph-23-00593-s001.zip › ijerph-4254602-supplementary.pdf]

Table S1: MINI Mpox vaccine recipients' insurance status by residential geography and clinical event Pride affiliation

Residential Geography	Total Recipients	Has Insurance (n = 1914)	Uninsured (n = 281)	Unknown (n = 64)
Urban	1105	928 (84%)	142 (12.8%)	35 (3.2%)
Out of State Urban	24	22 (91.7%)	2 (8.3%)	0
Metro	622	570 (91.6%)	40 (6.4%)	12 (2%)
Out of State Metro	47	36 (76.6%)	11 (23.4%)	0
Rural	146	132 (90.4%)	13 (8.9%)	1 (0.7%)
Out of State Rural	35	27 (77.1%)	7 (20%)	1 (2.9%)
Missing/Indeterminate	280	199 (71.1%)	66 (23.6%)	15 (5.3%)
**Residential Geography, Combined**				
All Urban Settings	1129	950 (84.1%)	144 (12.8%)	35 (3.1%)
All Metro Settings	669	606 (90.6%)	51 (7.6%)	12 (1.8%)
All Rural Settings	181	159 (87.8%)	20 (11.1%)	2 (1.1%)
**Residential Geography, Combined x Pride Event Affiliation**				
All Urban Settings x Pride	455	402 (88.4%)	45 (9.9%)	8 (1.7%)
All Metro Settings x Pride	521	476 (91.4%)	40 (7.7%)	5 (0.9%)
All Rural Settings x Pride	169	149 (88.2%)	19 (11.2%)	1 (0.6%)
All Urban Settings x non-Pride	674	548 (81.3%)	99 (14.7%)	27 (4%)
All Metro Settings x non-Pride	148	130 (87.9%)	11 (7.4%)	7 (4.7%)
All Rural Settings x non-Pride	12	10 (83.3%)	1 (8.3%)	1 (8.3%)

Internal
